# An Update on the Effectiveness of Probiotics in the Prevention and Treatment of Cancer

**DOI:** 10.3390/life12010059

**Published:** 2022-01-02

**Authors:** Vidya Sankarapandian, Balu Alagar Venmathi Maran, Ramya Lakshmi Rajendran, Manasi P. Jogalekar, Sridharan Gurunagarajan, Rajapandiyan Krishnamoorthy, Prakash Gangadaran, Byeong-Cheol Ahn

**Affiliations:** 1 Department of Microbiology, Srimad Andavan Arts and Science College, Bharathidasan University, Trichy 620005, India; sne777@andavancollege.ac.in; 2 Borneo Marine Research Institute, Universiti Malaysia Sabah, Kota Kinabalu 88400, Malaysia; bavmaran@ums.edu.my; 3 Department of Nuclear Medicine, School of Medicine, Kyungpook National University, Daegu 41944, Korea; ramyag@knu.ac.kr; 4 Helen Diller Family Comprehensive Cancer Center, University of California San Francisco, San Francisco, CA 94158, USA; manasi.jogalekar@ucsf.edu; 5 Department of Biochemistry, Srimad Andavan Arts and Science College, Bharathidasan University, Trichy 620005, India; srig@andavancollege.ac.in; 6 Nanobiotechnology and Molecular Biology Research Lab, Department of Food Science and Nutrition, College of Food and Agriculture Sciences, King Saud University, Riyadh 4545, Saudi Arabia; rkrishnamoorthy@ksu.edu.sa; 7 BK21 FOUR KNU Convergence Educational Program of Biomedical Sciences for Creative Future Talents, Department of Biomedical Science, School of Medicine, Kyungpook National University, Daegu 41944, Korea

**Keywords:** probiotics, immunomodulation, metastasis, biotherapy, oncogene kinase

## Abstract

Probiotics are living microbes that play a significant role in protecting the host in various ways. Gut microbiota is one of the key players in maintaining homeostasis. Cancer is considered one of the most significant causes of death worldwide. Although cancer treatment has received much attention in recent years, the number of people suffering from neoplastic syndrome continues to increase. Despite notable improvements in the field of cancer therapy, tackling cancer has been challenging due to the multiple properties of cancer cells and their ability to evade the immune system. Probiotics alter the immunological and cellular responses by enhancing the epithelial barrier and stimulating the production of anti-inflammatory, antioxidant, and anticarcinogenic compounds, thereby reducing cancer burden and growth. The present review focuses on the various mechanisms underlying the role of probiotics in the prevention and treatment of cancer.

## 1. Introduction

The term “probiotics” has a Greek origin and it literally means “for Life” [1]. The term was coined by Lilley and Stillwell in 1965 [2]. Fermented products, such as cheese, bread, wine, beer, and kefir, were widely used for their nutritional and therapeutic benefits long before the identification of probiotics [3]. Elie Metchnikoff, a Nobel laureate, was the first scientist to describe probiotics. He hypothesized that manipulating the intestinal microbiome with host-friendly bacteria would confer health benefits and delay senility [4]. He also considered *Lactobacillus* as a probiotic [1]. Hence, the credit of pioneering probiotics research is eternally devoted to Elie Metchnikoff. The Food and Agricultural Organization (FAO) defines probiotics as “live microorganisms which, when consumed in adequate amounts, confer beneficial health effects on the host” [5]. The human gastrointestinal tract is a reservoir of a complex and dynamic population of microorganisms (the gut microbiota), which mainly comprises bacteria (in number over 1014) and exerts a significant influence on the host during homeostasis and disease. The presence of such an abundance of intestinal bacteria contributes to the human body, having approximately ten times more prokaryotic cells than eukaryotic cells [6]. In recent years, studies on the use of probiotics for the prevention and treatment of human diseases have gained momentum [7].

### 1.1. Characteristics of Probiotics

**Figure 1:**
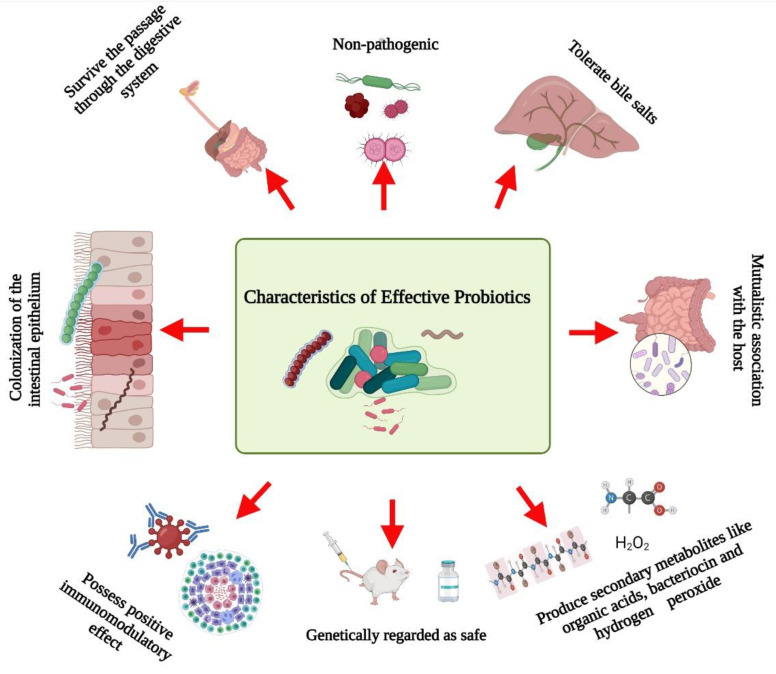
Schematic representation of the characteristics of effective probiotics. Created with BioRender.com, accessed on 28 December 2021.

Several researchers have documented the characteristics microorganisms should have to be effective probiotics (Figure 1). Briefly, probiotics:

✓ Are non-pathogenic [8];
✓ Can survive passage through the digestive system [9];
✓ Can tolerate bile salts [10];
✓ Are able to colonize the intestinal epithelium [11];
✓ Can maintain a mutualistic association with the host [12];
✓ Possess positive immunomodulatory effects [13];
✓ Are genetically safe [5];
✓ Produce beneficial metabolites such as organic acids, bacteriocin, and hydrogen peroxide [14].

### 1.2. Sources and Types of Probiotics

Several kinds of probiotics have been identified from different sources during the past decade. According to Śliżewska et al. [6], an organism identified as a probiotic usually belongs to the genera of bacteria or fungi, including *Lactobacillus, Pediococcus, Propionibacterium, Leuconostoc, Streptococcus, Enterococcus, Bifidobacterium, Bacillus, Saccharomyces cerevisiae, Candida pintolopesii, Aspergillus oryzae,* and *Aspergillus niger*. Lactic acid bacteria (LAB) are one of the most widely used probiotics [15]. Among LAB, *Bifidobacterium* and *Lactobacillus* are the most important microbes [16]. These probiotic microorganisms are isolated from different sources [6], such as vegetables, beef, salted crab, seafood, soybeans, yogurt, cheese, milk, kefir, human breast milk, barley, oat groats, molasses, grains, marine fish, smoked salmon, cabbage, wheat flour, sourdough, dairy products, chicken crop, porcine, and mangroves.

## 2. Probiotics and Cancer

**Figure 2:**
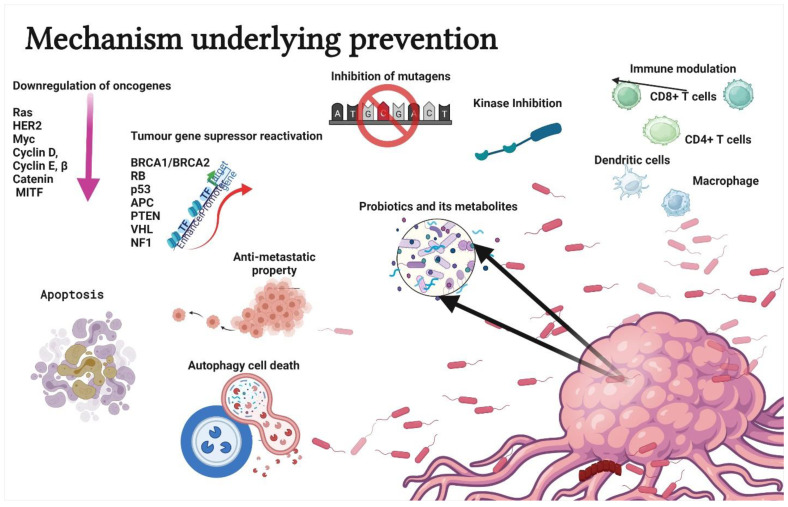
Schematic representation of mechanisms underlying the prevention or treatment of cancer using probiotics. The strategies include apoptosis, antimutagenic activity, down regulation of oncogene expression, autophagy induction in tumor cells, kinase inhibition, immune modulation, tumour gene suppressor reactivation, and antimetastatic property. Created with BioRender.com, accessed on 28 December 2021.

**Figure 3:**
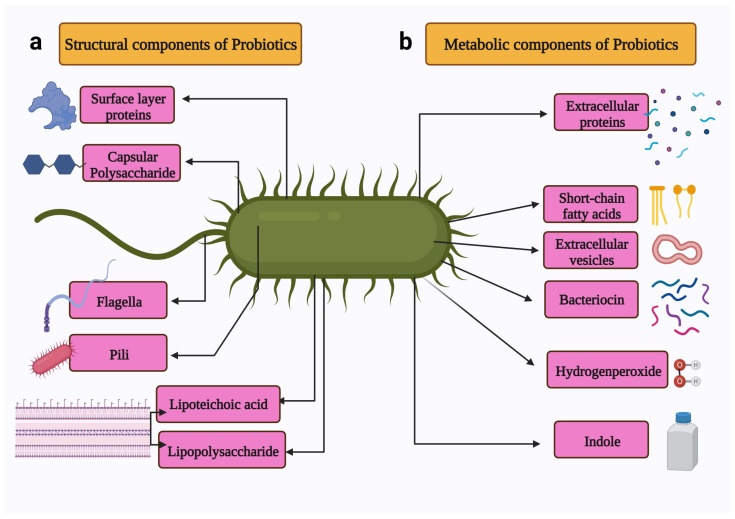
Schematic representation of the metabiotics of probiotics. The metabiotics of probiotics can be divided in two types: (**a**) Structural components include surface proteins, capsular polysaccharide, flagella, pili, lipoteichoic acid, and lipopolysaccharide. (**b**) Metabolic components include extracellular proteins, short-chain fatty acids, extracellular vesicles, bacteriocin, and indole. Created with BioRender.com, accessed on 28 December 2021.

Probiotics are used to treat several health conditions, such as dermatitis, inflammation, halitosis, diarrhoea, irritable bowel syndrome, hypercholesterolemia, obesity, urogenital infections, and cancers [17]. In particular, probiotics have gained attention due to their ability to modulate cancer signalling (Figure 2) [18]. Notably, probiotics can modulate cancers via the (a) induction of apoptosis [19], (b) inhibition of mutagenic activity [20], (c) downregulation of oncogene expression [21], (d) induction of autophagy [22,23], (e) inhibition of kinases [24], (f) reactivation of tumour suppressors [25], and (g) prevention of metastasis [26]. The anticancer properties of probiotics are mainly attributed to metabiotics (Figure 3). The term “metabiotics” refers to the structural components of probiotic microorganisms, their metabolites, and signalling molecules having a defined chemical structure that can optimize host-specific physiological functions as well as regulatory, metabolic, and behaviour reactions associated with the activity of the host [27].

The structural components of probiotics include surface layer proteins, capsular polysaccharides, flagella, pili, lipoteichoic acid, and lipopolysaccharides. These structural components constitute microbe-associated molecular patterns [28]. The metabolites produced by the probiotics include secreted proteins (extracellular proteins), hydrogen peroxide, indole, extracellular vesicles, short-chain fatty acids, and bacteriocins [29].

## 3. Role of Probiotics in Apoptosis Induction

**Figure 4:**
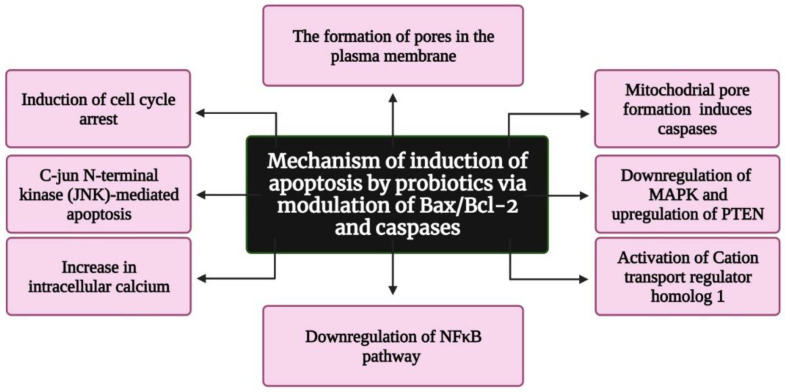
Flowchart representing the mechanism of apoptosis induction by probiotics via the modulation of Bax/Bcl-2 and caspases. Bax: Bcl-2-associated X protein; Bcl-2: B cell lymphoma 2; MAPK: mitogen-activated protein kinase; PTEN: Phosphatase and TENsin homolog deleted on chromosome 10; and NFkB: nuclear factor kappa-light-chain-enhancer of activated B cells. Created with BioRender.com, accessed on 28 December 2021.

Several reports suggest that probiotics inhibit tumorigenesis and cancer progression via apoptosis, but only a few studies have deduced the exact mechanism underlying apoptosis induction. According to Pfeiffer and Singh (2018), apoptosis is a promising target for cancer therapy [30]. Apoptosis is a form of cell death in which a “suicide” program is triggered, leading to DNA fragmentation, cytoplasm reduction, membrane changes, and cell death without lysis or damaging neighbouring cells [31]. The inhibition of tumour growth is one of the main functions of apoptosis [32]. Three interconnected pathways—mitochondrial/intrinsic pathway, death receptor/extrinsic pathway, and perforin/granzyme pathway—are involved in apoptosis [33]. The genes involved in apoptosis are tumour necrosis factor (TNF), inhibitors of apoptosis proteins, caspases, B cell lymphoma (Bcl)-2, and p53 gene [34]. Several reports indicate that probiotics induce apoptosis in cancer cells by modulating Bax/Bcl-2 and caspases [35,36] (Figure 4). In addition, colicin, a bacteriocin isolated from *Escherichia coli*, was found to have anticancer activity, resulting in the formation of minute pores on the plasma membrane [37]. These pores induce apoptosis and cause cell cycle arrest in the G1 phase. Preet et al. demonstrated the synergistic effect of nisin in combination with doxorubicin. They found that nisin and doxorubicin reduce the tumour volume by 66.82% in mice when compared with the untreated control [38]. Konishi et al. [39] analysed the probiotic-derived tumour-suppressive molecule ferrochrome, which has the ability to inhibit colon cancer progression via c-jun N-terminal kinase (JNK)-mediated apoptosis. Moreover, conjugated linoleic acid, a functional lipid produced by *Lactobacillus plantarum* (LPCLA), mediated apoptosis in breast cancer cells via the downregulation of the NFκB pathway [40]. *Lactobacillus acidophilus* and *Bifidobacterium bifidum* showed increased cytotoxic effects against breast and colon cancer cell lines by upregulating Bax, IFN-γ, and TNF-α expression and downregulating Bcl2 expression [41]. Further *L. acidophilus* induces apoptosis by increasing the mRNA expression of survivin and decreasing the mRNA expression of SMAC [42]. *Lactobacillus casei* significantly increases the expression of the hBD-2 gene in the cancer colon cell line HT29 [43]. Joo et al. reported that nisin induced apoptosis and reduced proliferation in HNSCC cells by increasing intracellular calcium, inducing cell cycle arrest, and activating cation transport regulator homolog 1 (Chac1) [44]. Jan et al. found that mitochondrial pore formation pathways induce apoptosis through caspase activation. Additionally, a study revealed that *Propionibacterium* caused apoptosis in colorectal carcinoma cells via the action of short-chain fatty acids on the mitochondria [45]. Overall, researchers continue to explore the apoptotic potential of probiotics on cancers. The field of probiotics-induced apoptosis research is rapidly progressing. Although many apoptotic proteins have been discovered, their molecular mechanisms of action largely remain unknown. 

Asoudeh-Fard et al. found that the probiotic *L. plantarum* induces apoptosis via the downregulation of mitogen-activated protein kinases (MAPK) and the upregulation of phosphatase and tensin homolog (PTEN) pathways [46]. Additionally, Zhang et al. reported that metabolites of *Lactobacillus spp.* have a negative effect on the viability of CAL-27 (human tongue squamous cell carcinoma) cells and induce apoptosis [47]. *L*. *salivarius* was found to reduce oral cancer in rats via the downregulation of COX-2/PCNA expression and the induction of apoptosis [48]. As the alteration in normal oral flora promotes oral cancer [49], the normal flora, particularly probiotics, plays a crucial role in the prevention of oral cancer [50].

## 4. Probiotics and Autophagy 

**Figure 5:**
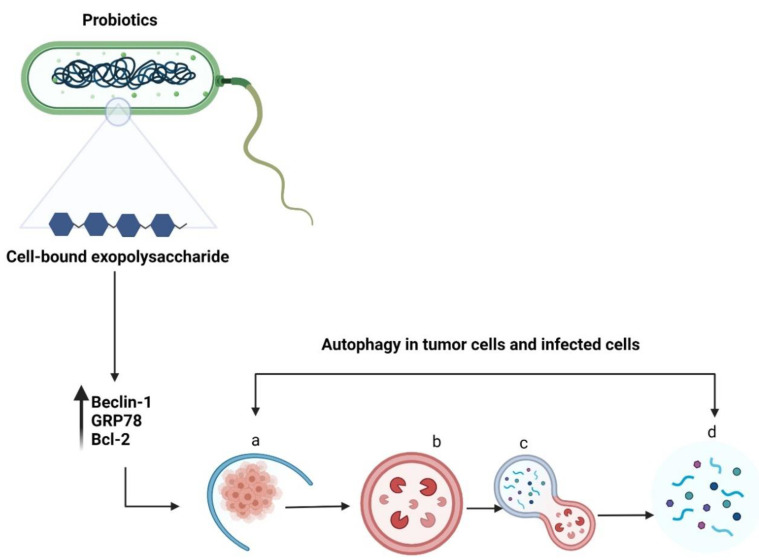
Schematic representation of autophagy in tumour cells or infected cells. The cell-bound exopolysaccharide of probiotic bacteria induces autophagy by upregulating *Beclin-1*, *GRP78*, and *Bcl-2* genes. The sequential events occurring during autophagy include (**a**) sequestration (**b**) transport to lysosomes, (**c**) degradation, and (**d**) utilization of degradation products. Created with BioRender.com, accessed on 28 December 2021.

Autophagy is a self-degradation process in which double-membrane autophagosomes sequester organelles or portions of the cytosol fuse with lysosomes for breakdown by resident hydrolases [51]. Although autophagy is essential for maintaining homeostasis in normal cells, it has also been implicated in various diseases. Increasing evidence suggests that autophagy promotes both tumour suppression [52] and progression [53]. Autophagy is vital for the elimination of damaged cells or aged proteins and organelles. Additionally, autophagy defects may lead to DNA damage and cancer, suggesting their role in tumour suppression [54]. Literature on the inhibition of tumour growth using probiotics via autophagy induction is lacking. Wang et al. reported that a surface protein from *L. acidophilus* induced HCT116 cell death by altering the levels of an autophagy-linked protein—microtubule-associated protein 1 light chain 3 (Figure 5) [55]. In addition, LAB promoted apoptosis induction in the presence of 5-fluorouracil by triggering Beclin1/GRP78-mediated autophagy activation [6]. The cell-bound exopolysaccharide of probiotics can potentially activate autophagy in colon cancer cells by stimulating Beclin1/GRP78 and the core regulators of intrinsic apoptosis pathway—Bcl-2 and Bak proteins [22]. The sequence of steps involved in autophagy are (a) sequestration (b) transport to lysosomes, (c) degradation, and (d) utilization of degradation products [56]. The important genes and proteins involved in autophagy include Beclin-1, lysosome-associated membrane protein, damage-regulated autophagy modulator 1, and p53 [57]. There are four different types of autophagy: chaperone-mediated autophagy, selective autophagy, macroautophagy, and microautophagy [58]. Autophagy can either promote or inhibit tumour development depending on several factors, such as cancer type or age [57]. However, probiotics-mediated autophagy and its role in the elimination of cancer warrants further investigation.

## 5. Probiotics as Potential Antimutagens

There are numerous agents that can cause DNA damage and mutations, which eventually lead to cancer [59]. Such agents that cause mutations are called mutagens. Carcinogenicity and mutagenicity are closely associated with each other [60]. Chemicals, ionizing and nonionizing radiations, and viruses are the widely known mutagens that cause cancers [61]. Probiotics are potential antimutagenic agents owing to their metabolites [62]. A study investigated the antimutagenic effects of probiotics against the mutagens sodium azide and benzopyrene and reported that probiotics have a binding potential for mutagens and are detoxifying antimutagens [63]. Of late, researchers are exploring the potential of probiotics as an alternate preservative and detoxifying agent [64,65,66]. The antimutagenic activity of *Lactobacillus rhamnosus* against the mutagen acridine orange has been previously analyzed and confirmed [67]. Table 1 presents the mutagen, antimutagenic microorganisms, and the source from which they are isolated.

The antimutagenic effect of probiotics is well-documented in the literature. The components of the probiotic cell wall, such as carbohydrates, proteins, lipids, and teichoic acids, are responsible for binding to the mutagens, and this interaction is hydrophilic [67,82]. Apart from the cell wall components, the glycoproteins secreted extracellularly [84] and organic acids, such as acetic, butyric, lactic, and pyruvic acids [20], also exhibit antimutagenic properties. For this reason, probiotics are used for the detoxification of food items and the treatment of some gastrointestinal disorders [85].

## 6. Probiotics-Mediated Tumour Suppressor Reactivation

Tumour suppressors slow down cell division, repair damaged DNA, and regulate apoptosis [86]. The tumour suppressor genes present in humans are *APC*, *BRCA1*, *BRCA2*, *p16*, *p21*, *p53*, *Rb*, and *VHL* [87,88]. Any defects or mutations in these genes can lead to cancers. Hence, it is crucial to reactivate tumour suppressor genes that are turned off by cancer cells. Many clinical trials are underway to determine if probiotics can be used as a potentially novel targeted biotherapy for cancers [89,90]. Sharma et al. demonstrated the involvement of short-chain fatty acids synthesized by probiotics in targeting tumour cells via the epigenetic regulation of the expression of tumour suppressor genes and oncogenes [91]. Epigenetic mechanisms alter gene expression without changing the primary DNA sequence [92]. Moreover, these mechanisms are heritable, reversible, and involve changes in DNA methylation, histone modifications, and small noncoding microRNAs (miRNAs) [92]. Metabiotics extracted from the probiotic *L. rhamnosus* MD inhibit colorectal cancer by upregulating the expression of the tumour suppressor gene p53 [93]. *L. rhamnosus* MD 14, *L. acidophilus*, and *L. rhamnosus* GG were shown to upregulate the expression of tumour suppressor genes in 1,2-dimethylhydrazine-induced experimental colon carcinogenesis model [94]. *Bifidobacterium longum*, isolated from breast milk, induced the expression of the tumour suppressor miRNAs miR-145 and miR-15 in murine colorectal cancer [95]. Kumar et al. emphasized that probiotic metabolites prevent colon cancer via epigenetic mechanisms and the metabiotics of probiotics play a key role in this process [96]. However, research related to the reactivation of tumour suppressors by probiotics is still in its infancy. 

## 7. Downregulation of Oncogene Expression by Probiotics

Oncogene expression causes cells to exhibit the properties of tumour cells, whereas proto-oncogenes are the normal nonmutated forms of oncogenes [97]. Proto-oncogenes are the precursors of oncogenes and are converted into oncogenes upon mutation [98]. The downregulation of oncogenes is one of the druggable targets of cancer therapy [99]. Several proto-oncogenes have been identified in different organisms by the virtue of structural homology to retroviral oncogenes [100]. The important proto-oncogenes in humans include *Ras, HER2, Myc, cyclin D, cyclin E, β-catenin*, and *MITF* (microphthalmia-associated transcription factor) [81]. Several reports have demonstrated the tumour-suppressive activity of probiotics via the downregulation of oncogenes [101,102]. The probiotic bacteria *Lactobacillus crispatus* and *L. rhamnosus* modulate cancers by altering the expression of mTOR-related genes and modulating the Wnt/β-catenin pathways [103]. Azam et al. showed that the culture supernatants of *L. acidophilus* and *L. crispatus* can downregulate cancer-testis gene *expression* in vitro [101]. A combination of probiotics and celecoxib (a nonsteroidal anti-inflammatory drug) can also downregulate the *KRAS* proto-oncogene, decreasing the incidence of colon cancer [94]. Understanding the mechanism of *KRAS* downregulation by probiotics could be beneficial for patients with *RAS*-associated cancers. Hosseini et al. revealed that the bacteriocin nisin as well as the cytoplasmic extract and cell wall of *Lactococcus lactis* decreased cyclin D1 expression, thereby inhibiting the proliferation of SW480 cells [104]. Lipoteichoic acid extracted from *L. plantarum* downregulated MITF [105]. Overall, approaches involving the regulation of oncogene expression using probiotics and their metabolites are being extensively investigated. 

## 8. Role of Probiotics in Preventing Metastasis

**Figure 6:**
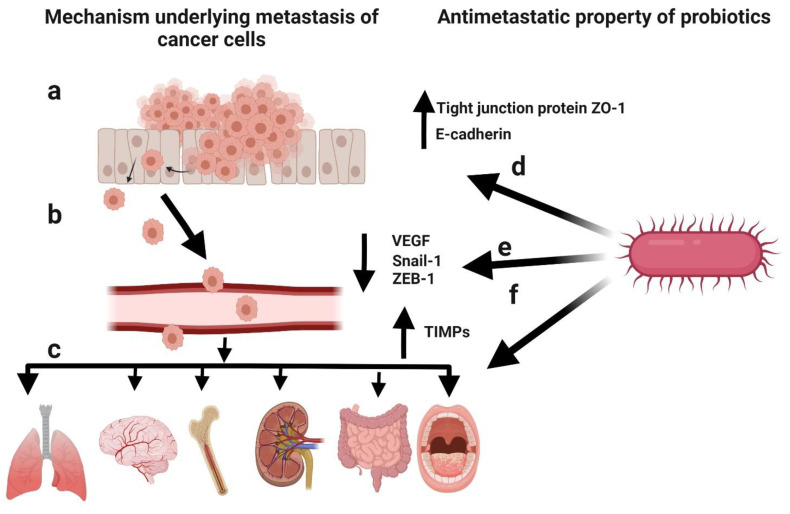
Schematic representation of the mechanism underlying cancer metastasis and the antimetastatic potential of probiotics. (**a**) Infiltration of cancer cells through the basement membrane. (**b**) Intravasation into the surrounding vasculature or lymphatic system. (**c**) Extravasation to secondary tissue and colonization as secondary tumours. (**d**) Elevated levels of the tight junction protein ZO-1 and E-cadherin induced by probiotics to inhibit metastasis. (**e**) Decreased levels of epithelial–mesenchymal transition (EMT)-related markers (Snail-1 and ZEB-1) and vascular endothelial growth factor (VEGF) induced by probiotics to inhibit metastasis. (**f**) Upregulation of tissue inhibitors of matrix metalloproteinases (TIMPs) by probiotics to inhibit metastasis. Created with BioRender.com, accessed on 28 December 2021.

Metastasis involves the detachment of tumour cells from the primary tumour and their dissemination to other parts of the body [106]. Cancer patients can develop metastasis years after the diagnosis of the primary tumour [107]. Metastasis is the cause of death in >90% of cancer patients [108]. It mainly occurs due to the epithelial–mesenchymal transition (EMT) of cancer cells [109], a physiological process by which epithelial cells attain the characteristics of mesenchymal cells, both physiologically and morphologically [110]. The metastasis of cancer cells can be divided into five steps: (1) infiltration of the basement membrane; (2) intravasation into the surrounding vasculature or lymphatic system; (3) persistence in the circulation; (4) extravasation to secondary tissue; and (5) colonization at secondary tumour sites [111]. The prevention of initial metastasis is crucial for improving the prognosis of cancer patients. Additionally, the inhibition of additional metastases in patients with metastases is helpful for improving the prognosis [112]. Several reports have highlighted the critical factors involved in metastasis, such as the interruption of cell–cell adhesion, EMT, tumour microenvironment, and cancer stem cell maintenance, as well as the antimetastatic effects of probiotics (Figure 6) [26]. Cell-free supernatants of probiotic *L. casei* and *L. rhamnosus* GG reduced the incidence of colon cancer as well as its metastatic effects by decreasing the levels of matrix metalloproteinase-9 (MMP-9) and increasing the levels of tight junction protein ZO-1 [113,114]. Additionally, the cell-free supernatant of *L. plantarum* YYC-3 inhibited the metastasis of colon cancer cells by suppressing the vascular endothelial growth factor (VEGF)-MMP2/9 signalling pathway [115]. VEGF is a signalling protein that promotes the growth of new blood vessels [116], whereas matrix metalloproteinases (MMPs) degrade the extracellular matrix [117]. Therefore, the suppression of the VEGF-MMP2/9 signalling pathway can inhibit the degradation of the basement membrane, which is the first step in metastasis. E-cadherin is the most important protein for cell–cell adhesion [118]. Li et al. observed a significant upregulation in E-cadherin levels in HeLa cells and the inhibition of cancer cell migration in response to probiotic treatment [119]. Additionally, probiotic treatment lowered the expression of EMT-related markers (Snail-1 and ZEB-1) in pancreatic cancer mouse models [120]. Kefir, a probiotic fermented food, showed antimetastatic and antiangiogenic effects in murine breast cancer cells, leading to the upregulation of tissue inhibitors of MMPs (TIMPs) [121]. Hence, probiotics play a key role in preventing metastasis.

## 9. Kinase Inhibition by Probiotics

Kinases and phosphatases are enzymes that add and remove phosphate groups, respectively [122]. Phosphorylation events alter other proteins by adding the terminal γ-phosphate group of adenosine triphosphate (ATP) to threonine, serine, and tyrosine residues [123]. Approximately 518 kinase-encoding and 156 phosphatase-encoding genes are estimated to be present in the human genome [124,125]. Kinases play a major role in various aspects of tumour biology, such as cell propagation, motility, metabolism, new blood vessel formation, and metastasis [126]. Hence, kinases are a potential therapeutic target for cancers [127]. A few studies have demonstrated the use of probiotics and their metabolites as kinase inhibitors for treating diarrhoea after cancer therapy [128,129]. Seth et al. demonstrated that probiotic secretory proteins protect the intestinal epithelial tight junctions and the barrier function from hydrogen peroxide-induced insult via a protein kinase C (PKC) and MAPK-dependent mechanism [130]. *L. plantarum* induces apoptosis by downregulating MAPKs and upregulating phosphatases [46]. *Lactobacillus* facilitated natural killer cell activity by producing tumour necrosis factor-associated apoptosis-inducing ligand, i.e., TNFAIL, in prostate cancer cell lines [131]. Further research efforts are targeting the kinase inhibitor activity of probiotics.

## 10. Bacteriocin as a Potent Anticancer Agent

A plethora of research has demonstrated the antioxidant and anti-inflammatory activities of metabiotics of probiotics, which forms the basis for their anticancer effects. Han et al. analysed the anti-inflammatory activity of *Lactobacillus lactis* NK34 strain in RAW 264.7 cells [132]. A significant reduction in the proliferation of cells and the production of nitric oxide and proinflammatory cytokines was observed [132]. The strains *Lactobacillus mucosae* AN1 and *Lactobacillus fermentum* SNR1 significantly reduced paw oedema and increased the expression of the anti-inflammatory cytokine IL-10 in comparison with the proinflammatory cytokine IL-6 [133]. Chooruk et al. reported that *L.* *fermentum, Lactobacillus paracasei,* and *L. rhamnosus* strains exhibit significant antioxidant activity [134]. Another study conducted by Yang et al. showed that probiotics downregulated the enzymes producing reactive oxygen species (ROS), glutathione (GSH), and butyrate [135]. Previous studies indicated that probiotics could produce folate [136,137] and bacteriocin, a low molecular weight protein with anti-inflammatory, anticancer, and immunomodulatory properties [138,139,140,141].

There are three major types of bacteriocins: class I (<5 kDa), class II (<10 kDa), and class III (>30 kDa) [142]. Bacteriocin is an FDA-approved compound that is commonly used in the food and pharmaceutical industry [143]. The anticancer activity of bacteriocin is well-documented in the literature [144,145,146]. Interestingly, bacteriocin specifically targets cancer cells and spares normal cells [147]. Normal cells are asymmetric in nature owing to the distribution of phospholipids on the inner and outer surfaces of the cell [148]. The outer layer of the normal cells is made up of sphingomyelin and phosphatidylcholine, which are neutral choline-containing zwitterionic phospholipids [149]. The inner layer is made up of phosphatidylserine and phosphatidylethanolamine, which are aminophospholipids [150]. On the contrary, cancer cells lack asymmetry as a result of changes in their phospholipids and carry a negative charge due to the presence of O-glycosylated mucins, heparin sulphates, and anionic phosphatidylserine [151]. Moreover, cancer cells have higher membrane fluidity and a number of microvilli compared with normal cells, resulting in an increased surface area [152]. Therefore, bacteriocin can preferentially bind to negatively charged tumour cells rather than neutrally charged normal cells [37]. Several studies have investigated cellular responses to bacteriocin in vitro (Table 2).

## 11. Drug Delivery Systems for Bacteriocin

**Figure 7:**
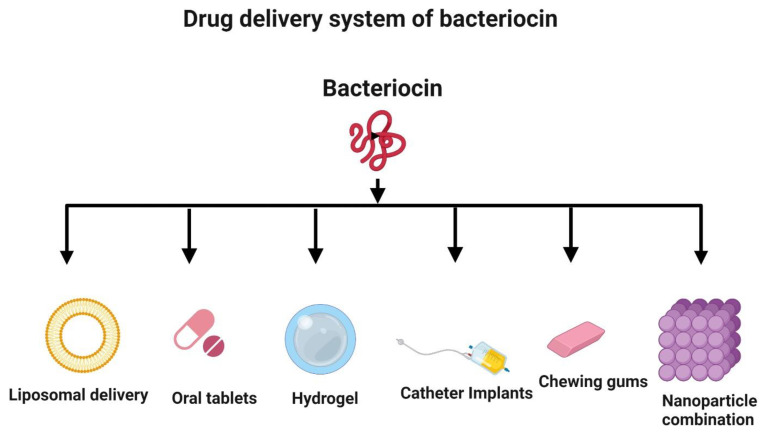
Novel delivery systems for bacteriocin. The strategies to deliver bacteriocin include liposomal delivery, oral administration (tablets and chewing gum), hydrogel embedding, medical device coating (e.g., catheter), and nanoparticle encapsulation. Created with BioRender.com, accessed on 28 December 2021.

Despite having excellent antimicrobial, antioxidant, and anticancer activities, bacteriocins may not be optimal for use as a drug delivery system. They can be easily digested by proteolytic enzymes in the intestinal tract. Hence, there is a need to examine alternative systems, such as liposomal delivery, for the delivery of antimicrobial and anticancer peptides (Figure 7) [155,156]. Nanotechnology is a valuable strategy to improve bacteriocin formulations and incorporate them into nanoparticles for delivery [157,158]. Nisin, a bacteriocin, has been successfully used in implants and delivered in vivo to prevent the growth of *Staphylococcus aureus* [159]. Hydrogels can be loaded with bacteriocins prior to delivery [155,160]. Bacteriocins can also be administered in the form of oral tablets [161] and chewing gum [162]. Additionally, bacteriocins can be used to coat medical devices, such as catheters, to prevent infections by inhibiting the adhesion of bacteria to their surfaces [163].

## 12. Clinical Trials with Probiotics

The World Health Organization defines clinical trial as “any research study that prospectively assigns human participants or groups of humans to one or more health-related interventions to evaluate the effects on health outcomes” [164]. While clinical trials investigating probiotics date back to the 1900s, the number of trials has increased significantly in recent years, with >100 studies being registered each year since 2010. According to ClinicalTrials.gov, accessed on 28 December 2021 and the International Clinical Trials Registry Platform, 323 and 1157 studies, respectively, are currently investigating the role of probiotics in improving oral health, gut microbiota, immune regulation, pH maintenance, and antimicrobial/anticancer activity throughout the United States, Europe, and Asia [165,166]. *L. rhamnosus* GG and *Bifidobacterium animalis* were the most frequently registered probiotic strains [167]. In addition, studies in children are higher in number than those in the elderly population according to ClinicalTrials.gov, accessed on 28 December 2021 [167]. A major limitation of the current studies is that many of them are observational. Nevertheless, immune regulation by probiotics has been demonstrated in studies investigating checkpoint inhibition as a potential anticancer therapy and colitis as an adverse effect of the therapy [167]. The ongoing research efforts are focused on studying the role of probiotics in treating gastrointestinal, metabolic, neurological, autoimmune, and communicable diseases [167]. Additionally, clinical trials have also highlighted the efficacy of probiotic strains in reducing the side effects of cancer-related microbiota dysbiosis [168]. More clinical trials that are inclusive of diverse populations and have a good statistical power are warranted to further explore the potential of probiotics in improving human health.

## 13. Conclusions and Future Perspectives

Probiotics have demonstrated efficacy (although variable, depending upon the strain, dosage, and duration of treatment) against various cancer types owing to their roles in antioxidation, immunomodulation, apoptosis induction, antimutagenicity, oncogene expression downregulation, autophagy induction, kinase inhibition, tumour suppressor reactivation, and metastasis prevention. A growing body of evidence suggests that probiotics can be used as an adjunctive therapy for cancer patients receiving chemotherapy. Although these findings are promising, large-scale randomized controlled trials are needed to determine the overall safety and efficacy of the formulations in treating cancer. Any regulatory issues and potential risks should also be addressed. The identification of specific probiotic strains that have the most benefits and minimal or no adverse effects in the context of cancer will be an important milestone in the development of a personalized approach for each patient with cancer. Probiotics induce tumour cell apoptosis and inhibit tumour cell proliferation and metastasis. However, considering that most of the current research on probiotics and cancer is limited to gastrointestinal tumours, the specific mechanism of probiotics against tumours has not been fully elucidated. As such, the therapeutic effects of probiotics must be carefully considered. As additional supplementary active microorganisms, the adverse reactions of probiotics, gastrointestinal side effects, skin reactions, access to antibiotic resistance genes, harmful effects of probiotic metabolites, and abnormal stimulation of the immune system must be evaluated.

## Figures and Tables

**Table 1:** List of mutagens, antimutagenic probiotics, and sources of probiotics.

Mutagen	Antimutagenic Microorganism	Source	Reference
Sodium azide and benzopyrene	*Lactobacillus alimentarius DDL 48, Enterococcus faecium DDE 39, Bifidobacterium bifidum DDBA,* and *Lactobacillus reuteri DDL 19*	Goat milk	[63]
Acridine orange	*Lactobacillus rhamnosus*	Infant feces	[67]
Heterocyclic amine binding and N-nitrosamine	*Lactobacillus plantarum CM4*	Thai fermented food products	[68]
Benzo[a]pyrene and sodium azide	*Lactobacillus* and *Bifidobacterium*	ATCC	[69]
4-nitro-O-phenylenediamine	*Lactobacillus acidophilus* and *Bifidobacteria*		[20]
Trp-P-1 and Trp-P-2	*Bifidobacterium longum*	Milk	[70]
Benzopyrene	*Bifidobacterium lactis Bb-12*, *Bifidobacterium longum CCRC 14634*		[71]
N-methyl-N’-nitro-N-nitrosoguanidine	*Lactobacillus, Streptococcus, Lactococcus,* and *Bifidobacterium*		[72]
N-methyl, N’-nitro, N-nitroso-guanidine, and 3,2’-dimethyl-4-amino-biphenyl	*Lactobacillus helveticus CH65, Lactobacillus acidophilus BG2FO4, Streptococcus salivarius ssp*., and *Lactobacillus delbrueckii sp. bulgaricus 191R*	Fermented milk	[73]
2-nitroflourene and nitroquinoline-1-oxide	*Lactobacillus paracasei subsp. tolerans JG22*	Pepper leaves Jangajji	[74]
N-methyl-N’-nitro-N-nitrosoguanidine	*Bifidobacterium breve* and *Bifidobacterium longum*	Human infant stool	[75]
4-nitro-O-phenylenediamine	*Lactobacillus plantarum KLAB21*	Kimchi (Korean fermented vegetables)	[76]
3-amino-1-methyl-5H-pyrido[4,3−b]indole (Trp-P2)	*Lactobacillus acidophilus LA106 (LA2)* and *Lactococcus lactis subsp. lactis L11103*	Milk	[77]
2-(2-furyI)-3-(5-nitro-2-furyl) acrylamide and 4-nitroquinoline-N-oxide	*Lactobacillus bulgaricus* and *Streptococcus thermophilus*	Milk	[78]
1,1-diphenyl-2-picrylhydrazyl and 2,2′-azino-bis(3-ethylbenzothiazoline-6-sulphonic acid)	*Lactobacillus acidophilus, Lactobacillus casei*, and *Lactobacillus paracasei subsp. paracasei*	Yogurt	[79]
Furazolidone	*Bifidobacterium lactis Bb-12* and *Lactobacillus acidophilus*		[80]
N-methyl-N0-nitro-N-nitrosoguanidine	*Lactobacillus rhamnosus*	Vaginal mucosa	[81]
Heterocyclic aromatic amines	*Lactobacillus helveticus*	Milk	[82]
Sodium azide (NaN3) and 2-nitrofluorene (2-NF)	*Lactobacillus plantarum*	Fermented durian	[83]

**Table 2:** Bacteriocins and their anticancer activity in select cell lines.

Bacteriocin	Source of Bacteriocin	Cell Lines	Reference
Enterocin LNS18	*Enterococcus*	HepG2 (liver cancer)	[141]
LHH1	*Lactobacillus casei HZ1*	MGC803, HCT116, and C666-1 (multiple origins)	[153]
Microcin E492	*Klebsiella pneumoniae*	HeLa (cervical cancer)	[154]
Laterosporulin10	*Brevibacillus sp. strain SKDU10*	MCF-7, HEK293T, HT1080, HeLa, and H1299 (multiple origins)	[141]

## Data Availability

Not Applicable.
